# A fast fish swimming protocol that provides similar insights to critical swimming speed

**DOI:** 10.1242/bio.060543

**Published:** 2024-08-12

**Authors:** Stephanie M. Bamford, Frank Seebacher

**Affiliations:** School of Life and Environmental Sciences A08, University of Sydney, Sydney, NSW 2006, Australia

**Keywords:** Acclimation, Performance, Repeatability

## Abstract

Performance measures are an important tool to assess the impact of environmental change on animals. In fish, performance is often measured as critical swimming speed (U_crit_), which reflects individual maximal physiological capacities. A drawback of U_crit_ is that trials are relatively long (∼30-75 min). U_crit_ may therefore not be suitable for several repeated measurements because of the potential for training effects, long recovery periods, and low throughput. Here we test a shorter (∼4-5 min) protocol, “U_crit_ fast” (U_Cfast_) in zebrafish (*Danio rerio*). We show that U_Cfast_ and U_crit_ have similar, significant repeatabilities within individuals. Unlike U_crit_, repeated U_Cfast_ trials did not elicit a training effect. Both U_Cfast_ and U_crit_ provide the same insights into thermal acclimation, and both provide similar estimates of individual acclimation capacity in doubly acclimated fish. We propose that U_Cfast_ is a valid substitute for U_crit_ particularly when higher throughput and several repeated measures are necessary.

## Teaser

**Summary:** We present a short protocol to measure swimming capacity in fish that can substantially increase throughput for complex experiments.

## INTRODUCTION

Performance may be defined as the value of a trait that is closely related to fitness, such as metabolic and locomotor traits (Husak et al., 2006; Le Galliard et al., 2004). The capacity to move fast enough to catch prey or long enough to disperse to new habitats will determine the success of animals in their natural environment (Denton et al., 2017; Domenici et al., 2019; Wu and Seebacher, 2022). Locomotor performance is therefore closely related to fitness, but it may also be costly so that there are trade-offs that determine individual fitness (Husak and Lailvaux, 2022). Environmental conditions can influence these responses and measures of locomotor performance are important to assess the impacts of environmental change on animals (Domenici et al., 2019; Killen et al., 2017, 2021). Temperature in particular has pronounced effects on locomotor performance acutely, and by eliciting acclimation responses (Claireaux et al., 2006; Sylvestre et al., 2007).

Locomotor performance is a widely used whole-animal performance measure in the literature, particularly in fish (Beamish, 1978; Nelson et al., 2002; Wu and Seebacher, 2022). The most common modes of locomotor performance measured are escape and sprint speed, and critical swimming speed (U_crit_) (Domenici and Blake, 1997; Kolok, 1999). Escape and sprint responses are linked to predator-prey interactions where they can determine escape success (Domenici et al., 2019). Sprints are classified physiologically as high-intensity movements that are powered anaerobically and last for less than 30 s, although escape responses in fish are much shorter than this (Burgomaster et al., 2008; Marras et al., 2013). In contrast, U_crit_ protocols are much longer (>30 min depending on context), and measure combined anaerobic and aerobic locomotor capacities (Marras et al., 2013; Plaut, 2001; Svendsen et al., 2010). Many fish species, including zebrafish, show positive rheotaxis, which reflexively orientates fish to swim into the water flow (Coombs et al., 2020) thereby facilitating forced-exercise tests such as U_crit_. U_crit_ tests in fish are similar to treadmill tests in humans and typically use swimming flumes in which fish swim at gradually stepped-up water flow speeds until a speed is reached where animals can no longer hold their position in the water flow (Plaut, 2001). U_crit_ is then calculated from the duration of each speed step and the time fish were able to swim at their final step. Actual flow speeds and the durations of steps vary between protocols and for different species (Hammer, 1995; Jain et al., 1997). In a typical protocol for zebrafish, water flow steps are 5-10 min in duration, and fish start the trial swimming at approximately 1-3 body lengths s^−1^ (0.03-0.1 ms^−1^), and at each step flow rate is increased by 1-2 body lengths s^−1^ (Seebacher and Bamford, 2024; Thambithurai et al., 2019). The total protocol lasts somewhere between 30 and 75 min depending on size, test temperature, and the thermal history of fish. U_crit_ depends on the combined capacities of the cardiovascular system, energy metabolism, and neuromuscular function and is therefore an excellent indicator of overall physiological performance capacity.

The length of U_crit_ trials are a drawback both in terms of the exercise intervention for the fish, and the time taken to complete a single run. Hence, the method has relatively low throughput, and may not lend itself to repeated measures because fish can need a relatively long time to recover from the exhaustive exercise (Zhang et al., 2018), and there is a likelihood of a training effect (Hammer, 1995; Simmonds and Seebacher, 2017). We therefore aimed to test a modified U_crit_ protocol (U_Cfast_), which reduces swimming time to around 5 min. The protocol reduces the duration of each speed step while keeping other parameters (e.g. magnitude of steps) the same. The rationale for the U_Cfast_ protocol is that anaerobically fuelled locomotion that relies primarily on fast muscle fibres is only of a very short duration (<30 s). Hence, a locomotor trial even lasting 5 min would rely on a combination of fast and slow muscle fibres and aerobic and anaerobic ATP production. We hypothesised, therefore, that a ∼5 min protocol should give similar responses to the much longer U_crit_ protocol. We tested this hypothesis in zebrafish (*Danio rerio*) by testing repeatability of U_crit_ and U_Cfast_, and comparing environmental effects on both measures of locomotor performance. We used temperature to test responses to environmental change because it is widely studied in the literature, has ubiquitous effects on physiological rate functions, and is relevant ecologically (Angilletta, 2009). Hence, we acclimated fish to different temperatures to measure acclimation responses and individual acclimation capacity to provide a range of different contexts for comparison between the two approaches.

## RESULTS AND DISCUSSION

### Repeatability

**Fig. 1.**
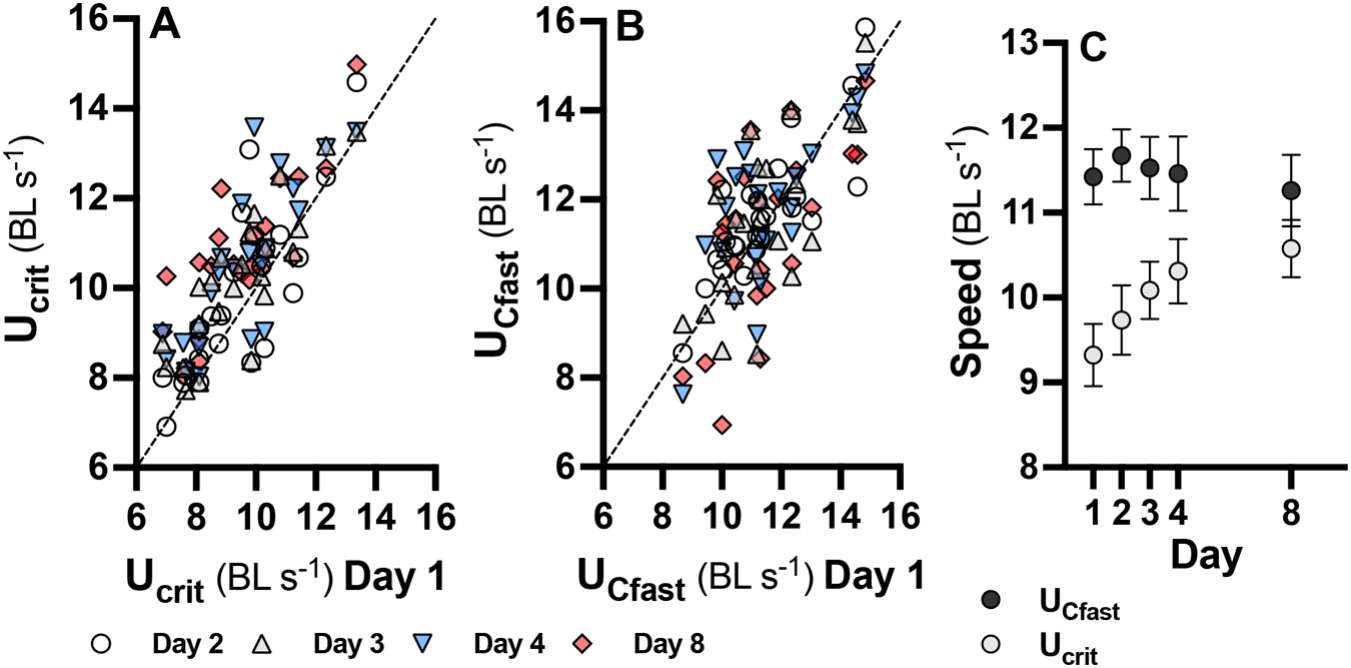
**Repeatability of swimming performance.** Both U_crit_ (A) and U_Cfast_ (B) were significantly repeatable (R=0.79 and 0.69, respectively) over 8 days [measurements taken on day 1 shown on x-axis, and days 2 (white circles), 3 (grey triangles), 4 (blue inverted triangles), and 8 (red diamonds) on y-axis], and repeatability did not differ between the two measures. Data from individual fish (*n*=24 for each U_crit_ and U_Cfast_) are shown. U_crit_ increased significantly with repeated swimming but U_Cfast_ did not (Fig. 1C); means±s.e. from all fish per day are shown.

Both U_crit_ [R=0.79 (95% confidence interval=0.63-0.87); *P*<0.001] and U_fast_ [R=0.69 (95% CI=0.51-0.81); *P*<0.001] were significantly repeatable (Fig. 1A,B). The broad overlap of confidence intervals indicates that repeatability did not differ between the two measures of swimming performance. Note that mean U_crit_ increased with repeated swimming (*P*=0.0018; Fig. 1C), but U_Cfast_ did not (*P*=0.98, Fig. 1C). These data indicate that either measure is a consistent representation of the intrinsic physiological performance of individual fish. However, the increase in performance following repeated U_crit_ trials likely represents a training effect that does not occur with the shorter exercise intervention of U_Cfast_ trials. Even though U_crit_ is repeatable across individuals, a training effect could introduce unwanted variation in cases where absolute performance (rather than relative performance between individuals) is of interest, such as in determining the time course of acclimation to a different temperature, for example.

### Thermal acclimation

**Fig. 2.**
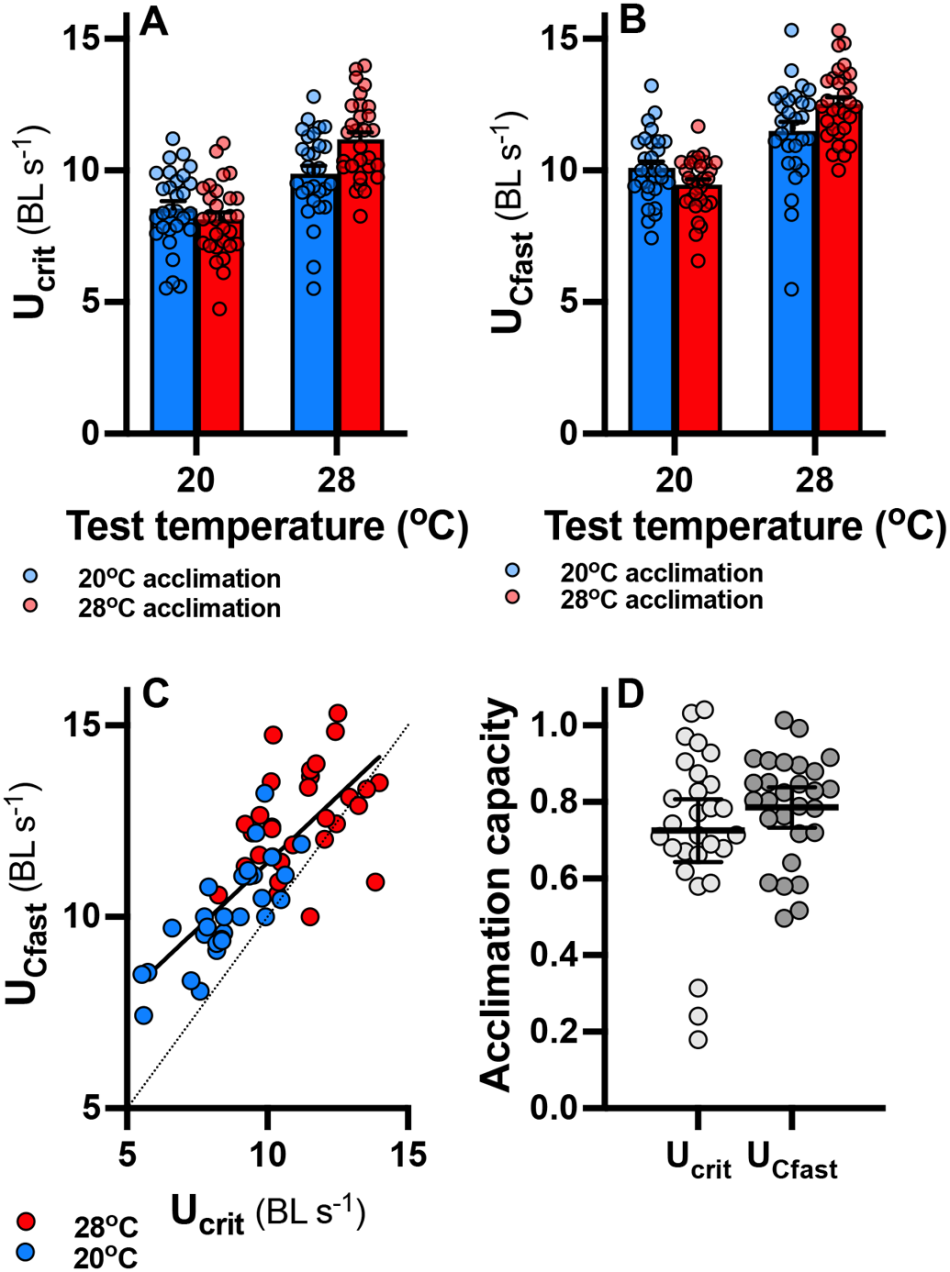
**Thermal acclimation of swimming performance.** Measurements of U_crit_ (A) and U_Cfast_ (B) responded similarly to acclimation to 20°C (blue bars) and 28°C and acute test temperatures of 20 and 28°C. In both cases, there were significant interactions between acclimation and test temperatures. Bars show means±s.e., and datapoints from individual fish are shown (*n*=28 for 20°C acclimation and *n*= 30 for 28°C acclimation). U_Cfast_ changed proportionally to U_crit_ across test temperatures (C; solid line, significant regression line; broken line, line of equality), but U_Cfast_ was somewhat higher particularly at cool temperatures. Fish were acclimated twice to determine individual acclimation capacity (D), which was not significantly different when based on U_crit_ or U_Cfast_; means (wide horizontal bars) ±95% confidence intervals as well as datapoints from individual fish are shown.

There were significant main effects of acclimation temperature (*P*<0.001), test temperature (*P*<0.001), and their interaction (*P*<0.0001). The interaction indicates that 28°C acclimated fish performed better at the higher test temperature (28°C) and, similarly, that 20°C acclimated fish performed better at 20°C test temperature compared to warm acclimated fish (Fig. 2A,B). There was a significant main effect of type of swimming performance, indicating that U_Cfast_ was overall higher that U_crit_, although the two measurements changed proportionally to each other across individuals and test temperatures (Fig. 2C, regression: Y=4.46+0.69x; R^2^=0.59, *P*<0.001). There were no other significant interactions (all *P*>0.80). Lack of significant interactions between types of swimming performance and any other fixed factors indicates that the two measures provide similar insights into thermal acclimation. U_crit_ or U_Cfast_ also gave similar individual acclimation capacity estimates derived from the double acclimation experiment (*P*=0.12; Fig. 2D). The three very low values in the U_crit_ data set reduced the mean acclimation capacity, which otherwise would have been identical to that from the U_Cfast_ data.

Aerobic and anaerobic pathways of energy (ATP) production are activated together during most exercise interventions. Lactate production, for example, increases in parallel with oxygen consumption (Warburg effect) as a normal part of exercise energetics in vertebrates, and reflects greater glycolytic flux (Poole et al., 2021). Nonetheless, the relative importance of anaerobic ATP production changes with duration of exercise. ATP supply of short (<30 s) bouts of intensive exercise is achieved by breakdown of stored phosphocreatine by creatine kinase, which is restored by anaerobic glycolysis and aerobic mitochondrial pathways (Barclay, 2017; Sahlin, 2014). As exercise duration increases and intensity decreases (i.e. longer sustained activity), the relative contribution of anaerobic pathways to restore phosphocreatine decreases (Barclay, 2017). Hence, our relatively more intense and shorter U_Cfast_ protocol may be energetically different from longer protocols such as U_crit_ because it is likely that anaerobic pathways make up a relatively greater contribution to total ATP supply of U_Cfast_ (Barclay, 2017). It may be conceivable that these energetic differences influence how locomotor performance responds to environmental variation such as changes in temperature, because there may be differences in the thermal sensitivity between aerobic and anaerobic energy supply pathways. However, our data indicate that this is not the case for zebrafish, and consistent use of a performance measures across treatments should ensure that treatment effects can be determined reliably.

The advantages of using the short U_Cfast_ protocol may be outweighed if the aim of an experiment is not just to determine a representative measure of swimming capacity but to measure an aspect of exercise physiology *per se*. For example, determination of aerobic cost of transport requires measurement of oxygen consumption while animals are moving at different speeds (Claireaux et al., 2006; Jahn and Seebacher, 2022). In this case, a U_crit_ protocol would be advantageous because it provides a longer window for oxygen consumption measurements and anaerobic processes are less important. U_Cfast_ and U_crit_ are by no means the only swimming protocols in the literature, and we summarised some of the different published protocols in Table 1. U_Cfast_ is most similar to a constant acceleration protocol (Farrell, 2008; Marras et al., 2013), where water flow is increased steadily until fatigue, except that swimming duration is still considerably longer in the latter. The main advantage of U_Cfast_ is its short duration, which is advantageous in cases where performance is measured repeatedly such as in time-course experiments. The shorter duration of U_Cfast_ also means that much higher throughput can be achieved in experiments, which opens the opportunity for larger sample sizes particularly in more complex factorial designs. Our result also show that U_Cfast_ provides similar insights into thermal acclimation as U_crit_, and both measures are equally repeatable. U_Cfast_ did not induce a training effect when implemented repeatedly, which can be advantageous because it does not introduce an additional source of variation. However, it may also mean that this protocol is not suitable for experiments that investigate the effects of exercise training *per se* or, at least, its suitability needs to be tested experimentally.

**Table 1.**
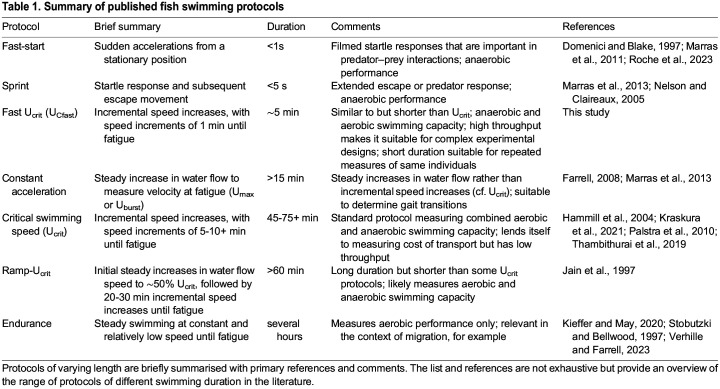
Summary of published fish swimming protocols

## MATERIALS AND METHODS

### Study animals

Adult shortfin zebrafish of mixed sex [*D. rerio*; mean standard length=3.28±0.02 (s.e.) cm; mean mass=0.56±0.014 (s.e.) g] were obtained from a commercial supplier (Livefish, Bundaberg, Australia). After arrival fish were dispersed across five tanks (0.65×0.28×0.32 m; 25 fish per tank) filled with filtered, aged water at 24°C until use in experiments after 1-2 weeks. During this holding period and in all experiments, water temperatures were maintained in a temperature-controlled room and each tank contained a biological filter and aerator. Water quality was monitored daily with test strips (API Fish Care, Australia) and 30% water changes were conducted twice weekly to maintain nitrates and nitrites at undetectable levels and pH between 6.8-7.0. Fish were fed daily with pellet food (O.range WEAN 2/4, Primo Aquaculture, Narangba QLD, Australia) supplemented with live *Artemia* twice per week. In handling fish, we minimised air exposure at all times, and before transfer to swimming flumes fish were kept individually in cylindrical plastic containers (1 l but filled with only ∼200 ml) and gently poured into swimming flumes. All procedures had the approval of the University of Sydney Animal Ethics Committee (approval #2021/1932).

### Swimming performance

We measured U_crit_ according to published protocols (Seebacher et al., 2015) in cylindrical, clear plastic (Perspex) flumes (150 mm length, 26 mm internal diameter) tightly fitted over the intake end of a submersible inline pump (12 V DC, iL500, Rule, Hertfordshire, UK). A plastic grid separated the flume from the pump, and a bundle of hollow straws at the inlet helped to maintain laminar flow. Flumes were submerged in plastic tanks (0.65×0.28×0.32 m), and we used a variable DC power source (NP9615; Manson Engineering Industrial, Hong Kong, China) to adjust the flow speed. Flow speed was measured in real-time during swimming trials using a flow meter (DigiFlow 6710M, Savant Electronics, Taichung, Taiwan) attached to the outlet of the pump. After transfer into the flume, fish swam at an initial flow rate of 0.1 m/s for 10 min followed by an increase in flow speed of 0.06 m/s every 10 min until the fish could no longer hold their position in the water column. The first time fish fell back to the plastic grid, flow was stopped for 10 s after which the previous flow rate was resumed. The trial was stopped when fish fell back to the grid a second time.

The U_Cfast_ protocol we tested here was identical to the U_crit_ protocol described above, but the duration of each speed increment was shortened and the second increment was used as the starting speed. Hence, fish were swum immediately after being placed in the flume at an initial speed of 0.16 m s^−1^ for 1 min, and speed was then increased by 0.06 m s^−1^ every 1 min. At 24°C (during repeatability tests, see below), the mean duration of U_crit_ trials was 49.6±0.6 (s.e.) min, and of U_Cfast_ trials it was 4.7±0.08 (s.e.) min. There were 24 h between swimming trials and each fish was swum only once per day.

### Repeatability

We repeatedly measured U_crit_ or U_Cfast_ in the same individuals, but only one method (U_crit_ or U_Cfast_) was measured in a given individual (*n*=24 fish each for U_crit_ and U_Cfast_). Fish were swum 1, 2, 3, and 8 days apart to determine whether swimming performance measures reflect consistent phenotypes. Trials were conducted at 24°C, and fish were dispersed across six glass tanks (0.30×0.18×0.20 m; four fish per tank) between trials. Between swimming trials fish were kept in perforated cylindrical baskets (1 l volume) within their home tanks so that we could follow individuals while still permitting visual and olfactory contact between fish (Seebacher et al., 2015). The experiment was conducted in two blocks with *n*=24 fish in each block. In half the fish within each block we measured U_crit_ and in the other half we measured U_Cfast_.

### Acclimation capacity

We measured acclimation and individual acclimation capacity (Seebacher et al., 2015) of fish (*n*=28) by acclimating each individual to warm and cold conditions sequentially. As above, individual fish were kept in perforated cylindrical plastic containers (1 l volume; in 0.30×0.18×0.20 m tanks; four fish per tank) during the acclimation period. Each fish was acclimated twice, with half the fish randomly assigned to the cool (20°C) acclimation treatment first for 3-4 weeks, and then to the warm (28°C) acclimation treatment for 3-4 weeks, and the other half to the opposite order to dilute any order effects. To reach the respective acclimation temperatures, water temperatures were changed gradually by 3-4°C per day over 2 days (Hammill et al., 2004). Acclimation temperatures were well within the natural range of temperatures experienced by zebrafish (López-Olmeda and Sánchez-Vázquez, 2011). During acclimation, fish were dispersed across four glass tanks (0.30×0.18×0.20 m) within each treatment. After each acclimation period, we measured U_crit_ and U_Cfast_ at 20°C and 28°C acute test temperatures in each individual; as with the temperature treatments above, the order of measurements was split and in half the fish per treatment we measured U_Cfast_ first and in the other half U_crit_ to dilute order effects. Fish were given at least 24 h to recover after each swimming performance measure. After each acclimation treatment, we photographed each fish to determine body length (using ImageJ software, National Institute of Health, USA). Fish were placed into tared weighing dishes filled with sufficient water to cover fish to be weighed on an electronic balance; to avoid injury, we did not blot the fish directly to remove excess water before placing them into weighing dishes, but removed as much water as possible while fish were held in a soft net.

Acclimating each individual to both temperatures allowed us to quantify phenotypic plasticity of each individual, expressed as an acclimation capacity index (Seebacher et al., 2015):

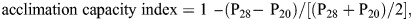
where P_28_ is the swimming performance of a fish that is acclimated to 28°C and measured at 28°C acute test temperature, while P_20_ is the equivalent measure at 20°C. The acclimation capacity index indicates relative thermal compensation (the ability to maintain constant performance across thermal conditions) by contrasting the difference between P_20_ and P_28_. Acclimation capacity approaches 1 as P_20_ approaches P_28_, and decreases as the difference between P_28_ and P_20_ increases. The index could also be expressed in the opposite direction as P_20_–P_28_, but fish tended to perform better in warm conditions due to the thermodynamic depression caused by cold temperatures. If a fish over-compensated for low temperatures and P_20_>P_28_, the index will exceed 1. The index is based on the difference between P_20_ and P_28_ normalised to their mean, and is therefore a dimensionless number that is independent from the absolute values of P_20_ and P_28_.

### Statistical analysis

We analysed repeatability of swimming performance of individuals between days of measurements in the R package rptR (Stoffel et al., 2017). Repeatability (R) represents the fraction of the total phenotypic variance in the population that can be attributed to individual identities, and significance is estimated by permutational *P*-values (Stoffel et al., 2017). We used permutational analyses of variance in the R package lmPerm (Wheeler and Torchiano, 2016) for the remainder of the analyses. Permutational analyses are advantageous because the data *per se* are used for analysis and no assumptions about underlying distributions are necessary; statistical results are given as permutational *P*-values (Drummond and Vowler, 2012; Ludbrook and Dudley, 1998). Differences in mean performance between repeated days of swimming were analysed with day as the fixed factor and individual ID as a random factor. In analyses of acclimation responses, acclimation temperatures, acute test temperatures, and swimming performance type (U_crit_ or U_Cfast_) were fixed factors. Swimming performance was analysed in m s^−1^ with standard length of fish as a co-variate, and we used individual ID as a random variable to account for repeated measures of individual fish. Individual acclimation capacity was analysed with swimming performance type as fixed factor and individual ID as random factor.
